# Tomosyn Inhibits Synaptic Vesicle Priming in Caenorhabditis elegans

**DOI:** 10.1371/journal.pbio.0040261

**Published:** 2006-07-25

**Authors:** Elena O Gracheva, Anna O Burdina, Andrea M Holgado, Martine Berthelot-Grosjean, Brian D Ackley, Gayla Hadwiger, Michael L Nonet, Robby M Weimer, Janet E Richmond

**Affiliations:** **1**Department of Biological Sciences, University of Illinois at Chicago, Chicago, Illinois, United States of America; **2**Biology Department, Loyola University Chicago, Chicago, Illinois, United States of America; **3**Department of Anatomy and Neurobiology, Washington University School of Medicine, Saint Louis, Missouri, United States of America; **4**Cold Spring Harbor Laboratory, Cold Spring Harbor, New York, United States of America; Princeton UniversityUnited States of America

## Abstract

Caenorhabditis elegans TOM-1 is orthologous to vertebrate tomosyn, a cytosolic syntaxin-binding protein implicated in the modulation of both constitutive and regulated exocytosis. To investigate how TOM-1 regulates exocytosis of synaptic vesicles in vivo, we analyzed *C. elegans tom-1* mutants. Our electrophysiological analysis indicates that evoked postsynaptic responses at *tom-1* mutant synapses are prolonged leading to a two-fold increase in total charge transfer. The enhanced response in *tom-1* mutants is not associated with any detectable changes in postsynaptic response kinetics, neuronal outgrowth, or synaptogenesis. However, at the ultrastructural level, we observe a concomitant increase in the number of plasma membrane-contacting vesicles in *tom-1* mutant synapses, a phenotype reversed by neuronal expression of TOM-1. Priming defective *unc-13* mutants show a dramatic reduction in plasma membrane-contacting vesicles, suggesting these vesicles largely represent the primed vesicle pool at the C. elegans neuromuscular junction. Consistent with this conclusion, hyperosmotic responses in *tom-1* mutants are enhanced, indicating the primed vesicle pool is enhanced. Furthermore, the synaptic defects of *unc-13* mutants are partially suppressed in *tom-1 unc-13* double mutants. These data indicate that in the intact nervous system, TOM-1 negatively regulates synaptic vesicle priming.

## Abstract 2

This paper examines the in vivo role of the syntaxin binding protein tomosyn in synaptic transmission at the *C. elegans* neuromuscular junction. Tomosyn inhibits vesicle priming by regulating the size of the readily releasable vesicle pool.

## Introduction

Membrane fusion is mediated by the interactions of cognate SNARE (soluble NSF attachment protein receptor) proteins associated with vesicle and target membranes [ 1, 2]. Synaptic vesicle exocytosis is a highly specialized form of membrane fusion in which calcium triggers fusion of synaptic vesicles with the plasma membrane, resulting in neurotransmitter release. Prior to vesicle fusion, the plasma membrane Q-SNAREs syntaxin-1a and SNAP-25 assemble with the vesicle-associated R-SNARE synaptobrevin-2 (a.k.a. VAMP-2) to form a stable coiled-coil complex known as the SNARE complex [ 3, 4]. The assembly of the SNARE complex in *trans* is thought to bring the vesicle into close apposition with the plasma membrane, and may drive the fusion reaction [ 5]. Several synaptic proteins have been implicated in the regulation of this fusion process through their SNARE interactions, including the recently identified protein tomosyn [ 6].

Tomosyn is a 130 kDa soluble protein first isolated from rat cerebral cytosol as a syntaxin-binding partner capable of disrupting Munc18–syntaxin-1a complexes [ 6]. There are two paralogous tomosyn genes in the mammalian genome (tomosyn-1 and −2) that give rise to seven tomosyn isoforms through differential splicing [ 7, 8]. All mammalian tomosyn isoforms have two recognizable domains, an N-terminal domain rich in WD40 repeats and a C-terminal SNARE domain with high sequence homology to the R-SNARE domain of synaptobrevin [ 9, 10]. WD40 repeats are known to form beta propellers that act as protein interacting modules, although binding partners of the tomosyn WD40 repeats have yet to be identified. The tomosyn R-SNARE domain interacts with syntaxin and SNAP-25 to form a tomosyn SNARE complex that does not contain synaptobrevin but still binds to the putative calcium sensor synaptotagmin [ 6]. The biophysical properties of the tomosyn SNARE complex resemble those of the SNARE complex: both form at similar rates, have strong hysteresis during folding/unfolding transitions, exhibit alpha helicity, and are disassembled by NSF [ 10]. Consistent with these properties, the crystal structure of the core tomosyn SNARE complex reveals a four-alpha helical arrangement between the SNARE domains of tomosyn, SNAP-25, and syntaxin that is similar to the structure of the synaptobrevin-containing SNARE complex [ 11]. As predicted from the similarity in crystal structures, synaptobrevin is unable to displace tomosyn from the tomosyn SNARE complex (and vice versa), without prior NSF disassembly [ 11]. These data imply that formation of tomosyn SNARE complexes may preclude synaptobrevin-containing SNARE complex assembly, and therefore negatively regulate vesicle exocytosis. Consistent with this model, overexpression of vertebrate tomosyn reduces depolarization-induced dense-core granule fusion from PC12 cells [ 6, 10], chromaffin cells [ 12], insulin-mediated exocytosis of GLUT4-containing vesicles from adipocytes [ 13], insulin release from beta cells [ 14], and synaptic transmission in cultured superior cervical ganglion neurons [ 15]. Although these data support a negative regulatory role for tomosyn in vesicle fusion, tomosyn RNA interference (RNAi) experiments have yielded mixed results. In cultured superior cervical ganglion neurons, tomosyn RNAi inhibited evoked release [ 15], whereas tomosyn RNAi in mouse beta cells enhanced exocytosis [ 14].

To address the role of tomosyn in synaptic transmission, in this study we directly assayed the physiological phenotype of tomosyn loss-of-function mutants at the Caenorhabditis elegans neuromuscular junction (NMJ). Our results indicate that tomosyn inhibits synaptic transmission through actions that regulate the size of the readily releasable vesicle pool.

## Results

### *tom-1* Encodes the C. elegans Tomosyn Homolog

**Figure 1:**
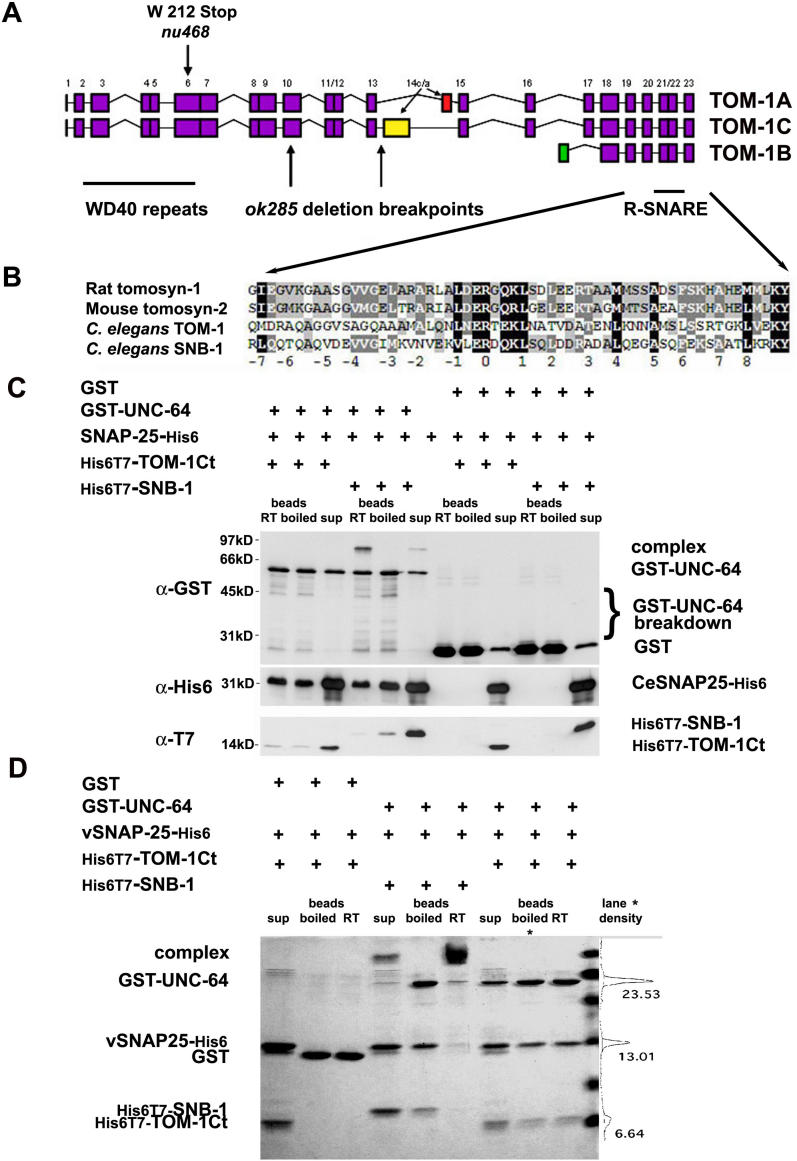
TOM-1 Is the C. elegans Tomosyn Homolog (A) Gene structure of the three C. elegans TOM-1 isoforms. All isoforms were confirmed by expressed sequence tags 3′ to the 6th exon and the upstream 5′ region common to TOM-1A, and TOM-1C was determined by 5′ RACE. The two long isoforms TOM-1A and TOM-1C contain N-terminal WD40 repeats and a C-terminal SNARE domain. The short isoform (TOM-1B) contains only the SNARE domain. *tom-1(nu468)* is a G to A change in W212 resulting in an early stop predicted to disrupt isoforms TOM-1A and TOM-1C [ 16]. The mutation in *tom-1(ok285)* is a 1,580-bp deletion that removes part of exon 10 and all of exons 11 through 13. *tom-1(ok285)* disrupts TOM-1A but, by RT-PCR can produce a mRNA capable of encoding an alternative isoform of TOM-1C lacking exons 11–13 and containing a partial exon 10 and an extra eight amino acids. (B) Amino acid alignment of the R-SNARE domain of rat tomosyn-1, mouse tomosyn-2, C. elegans TOM-1, and C. elegans synaptobrevin-1. Identity is shown as black boxes and similarity as gray boxes. Numbers below indicate helical layers formed during SNARE complex assembly. (C) TOM-1Ct forms tomosyn SNARE complexes with syntaxin and SNAP-25 in vitro to the same extent as C. elegans synaptobrevin (SNB-1) forms synaptobrevin SNARE complexes. C. elegans syntaxin::GST, or GST alone was incubated with SNAP-25–His6 and either His6T7-tagged TOM-1Ct, or His6T7-tagged SNB-1. Complexes were isolated using glutathione agarose beads. SDS resistance of complexes was assayed by heating half of the pull-down before electrophoresis. Proteins were separated on SDS-PAGE and transferred to nitrocellulose and detected with anti-GST, anti-His6, and anti-T7 antibodies. (D) Mixed-species tomosyn SNARE and synaptobrevin SNARE complexes form efficiently. Complexes were formed and isolated as described for (C) except that rat SNAP-25 (vSNAP-25–His6) was used in place of the C. elegans protein. Complexes were visualized by Coomassie Blue staining after SDS-PAGE. Complex stoichiometry of the tomosyn SNARE complex (*) was estimated by densitometry shown on left. The integrated density of each peak is listed. The molecular weight markers are (from top to bottom) 97 kDa, 66 kDa, 45 kDa, 31 kDa, 21 kDa, and 14 kDa.

The C. elegans genome encodes a single tomosyn gene [ 16], *tom-1*, that shares significant identity (˜33%) with isoforms of mammalian tomosyn-1 and tomosyn-2 ( Figure 1A). Like vertebrate tomosyn, *C. elegans tom-1* encodes multiple alternatively spliced isoforms, TOM-1(A,B,C) which share the C-terminal coiled-coil motif resembling the R-SNARE domain of C. elegans synaptobrevin (SNB-1) ( Figure 1B). The TOM-1B isoform is much smaller than either TOM-1A or TOM-1C, and lacks the N-terminal WD40 repeats. To examine whether the common C-terminal coiled-coil motif of TOM-1 (TOM-1Ct) interacts with syntaxin and SNAP-25, we performed in vitro pull-down assays using recombinant C. elegans proteins. We compared the behavior of synaptobrevin and TOM-1Ct in complex assembly assays with syntaxin-GST (UNC-64) [ 17] and SNAP-25 (RIC-4) [ 18]. Both SNB-1 and TOM-1Ct formed complexes, but did so inefficiently ( Figure 1C). Systematic replacement of each protein in the SNARE complex assay revealed that all assays containing the *C. elegans* SNAP-25 formed inefficiently, while all other mixed species formed complexes efficiently ( Figure S1). When the TOM-1Ct was mixed with C. elegans syntaxin-GST and vertebrate SNAP-25, syntaxin-GST co-precipitated TOM-1Ct and SNAP-25 ( Figure 1D). TOM-1Ct did not form a stable binary complex with syntaxin ( Figure S2). TOM-1Ct formed complexes as efficiently as SNB-1 forms complexes, but the TOM-1–containing complex was SDS sensitive, while the SNB-1–containing complex was SDS resistant. Density traces of the Coomassie blue–stained protein incorporated into the mixed-species TOM-1 complex gave densitometry ratios of 1:1.3:1.2 when divided by the molecular mass for each of the fusion proteins ( Figure 1D), consistent with the 1:1:1 stoichiometry reported for the vertebrate tomosyn complex [ 10]. These data confirm that C. elegans TOM-1 has the ability to form pseudo-SNARE complexes similar to those of vertebrate tomosyn.

### *tom-1* Mutant Behavioral and Electrophysiological Phenotypes

To investigate the function of TOM-1 in vivo, we characterized the phenotypes of two previously isolated tomosyn hypomorphic mutants, *tom-1(ok285)* and *tom-1(nu468),* which disrupt both full-length TOM-1 isoforms (TOM-1A/C) but are not predicted to disrupt the short TOM-1B isoform ( Figure 1A) [ 16]. These mutants are viable but exhibit hypersensitivity to the acetycholinesterase inhibitor aldicarb, suggesting that cholinergic neurotransmission is enhanced either pre- or postsynaptically [ 16]. In several behavioral assays *tom-1* mutants exhibit mild defects, including reduced brood size (331.5 ± 7.2 progeny, *n* = 10, for wild-type [WT] vs. 299.2 ± 14.4 progeny, *n* = 9, for *tom-1(ok285), p* = 0.055 *;* and 206.6 ± 11.8 progeny, *n* = 9, for *tom-1(nu468), p* < 0.0001), decreased thrashing rates in solution (121.7 ± 1 body bends/min, *n* = 10, for WT vs. 103.2 ± 3.9 body bends/min, *n* = 10, for *tom-1(ok285), p* = 0.0002; and 92.3 ± 2.4 body bends/min, *n* = 10, for *tom-1(nu468), p* < 0.0001), and altered responses to head taps: specifically, *tom-1* mutants exhibited increased forward/backward reversals (1.3 ± 0 .4 reversals, *n* = 7, for WT vs. 4.9 ± 1.5 reversals, *n* = 7, for *tom-1(ok285), p* = 0.036; and 4.1 ± 0.9 reversals, *n* = 7, for *tom-1(nu468), p* = 0.011) and increased incidence of pauses (0 pauses, *n* = 7, for WT vs. 4.4 ± 1.5 pauses, *n* = 7, for *tom-1(ok285)*; and 7.3 ± 2.2 pauses, *n* = 7, for *tom-1(nu468)*). *tom-1* mutants were indistinguishable from WT for other behaviors, such as defecation cycle (intervals between expulsion events: 53.4 ± 0.5 s, *n* = 5, for WT vs. 53.2 ± 2 s, *n* = 5, for *tom-1(ok285);* and 52.8 ± 1.3, *n* = 6, for *tom-1(nu468)).* The aldicarb hypersensitivity and behavioral changes of *tom-1* mutants are consistent with the hypothesis that TOM-1 function is required for proper signaling between neurons.

**Figure 2:**
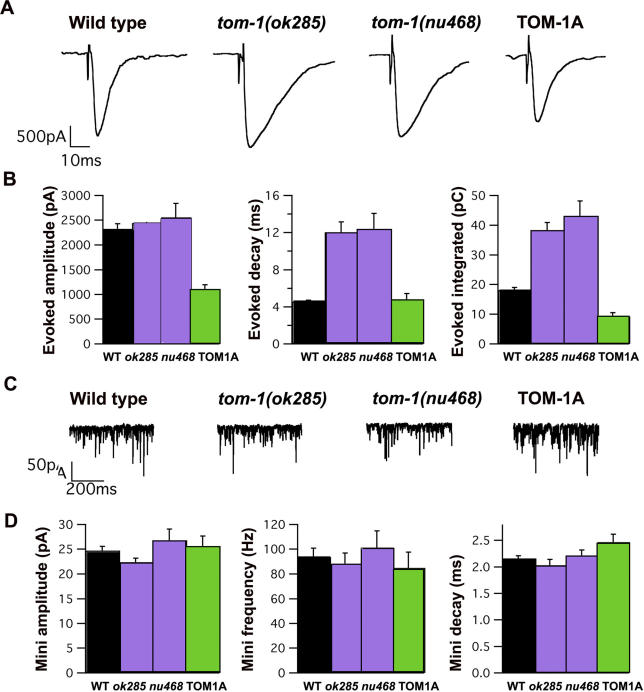
Electrophysiological Phenotypes of *tom-1* Mutants (A) Electrophysiological recordings from NMJs of dissected worms, revealed a pronounced increase in evoked response duration in both *tom-1* mutant alleles *(ok285* and *nu468)* that is reversed in *jaIs1052,* expressing TOM-1A in cholinergic neurons of *tom-1(nu468*) *.* (B) Analysis of evoked responses detected as inward currents from voltage-clamped body wall muscles in response to 2 ms depolarizing ventral cord stimuli. Evoked amplitude (pA) is normal in *tom-1* mutants and decreased in *jaIs1052* (left). Half-time–evoked response decay (ms) is increased in *tom-1* mutants and restored to normal levels in *jaIs1052* (middle). Total charge integral is increased in *tom-1* mutants and reduced in *jaLs1052* (right). (C) Representative endogenous miniature postsynaptic events. (D) Event amplitude (left), frequency (middle), and decay rates (right) were normal in *tom-1* mutants and *jaIs1052*.. Data expressed as mean ± SEM.

To directly test whether mutations in *tom-1* alter synaptic transmission, we recorded synaptic responses from the NMJs of dissected worms. Evoked responses were elicited by applying a depolarizing stimulus to the ventral nerve cord and recorded from voltage-clamped postsynaptic body wall muscles. In 5 mM Ca ^2+^ saline the evoked current amplitudes were not significantly altered in *tom-1* mutants (2,327 ± 107 pA, *n* = 47, for WT vs. 2,443 ± 136 pA, *n* = 37, for *tom-1(ok285), p* = 0.5; and 2,560 ± 283 pA, *n* = 11, for *tom-1(nu468), p* = 0.37) ( Figure 2A and 2B). However, the charge integral, a measure of the total ion flux during the evoked response (18.1 ± 0.97 pC, *n* = 49, for WT vs. 38.2 ± 2.7 pC, *n* = 30, for *tom-1(ok285), p* < 0.0001; and 42.6 ± 5.6 pC, *n* = 8, *tom-1(nu468), p* < 0.0001) ( Figure 2B), was greatly increased in *tom-1* mutants due to a prolonged postsynaptic response (half-time–evoked decay: 4.6 ± 0.17 ms, *n* = 47, for WT vs. 12 ± 1.1 ms, *n* = 30, for *tom-1(ok285), p* < 0.0001; and 12.3 ± 1.7 ms, *n* = 8, for *tom-1(nu468), p* < 0.0001) ( Figure 2B). The enhanced evoked charge integral of *tom-1* mutants in the absence of an increase in evoked amplitude could reflect saturation of the postsynaptic receptor field under the relatively high calcium (5 mM) recording conditions used. To test this possibility, we measured the evoked responses of *tom-1* mutants in 0.5 mM Ca *^2+^*. In lower calcium, the evoked response amplitudes of *tom-1* mutants were still comparable to the WT (1,100 ± 108 pA, *n* = 13, for WT vs. 1,410 ± 178 pA, *n* = 7, for *tom-1(ok285), p* > 0.1; and 1,533 ± 202 pA, *n* = 8, for *tom-1(nu468)*, *p* > 0.05); however, the response duration remained significantly prolonged (half-time decay: 2.7 ± 0.17 ms for WT vs. 4.2 ± 0.57 ms for *tom-1(ok285), p* = 0.016; and 4.2 ± .37 ms for *tom-1(nu468), p* = 0.0014), again resulting in increased charge integrals (6.9 ± 0.82 pC for WT vs. 10.7 ± 1.2 pC for *tom-1(ok285), p* = 0.018; and 13.3 ± 2 pC for *tom-1(nu468), p* = 0.006). These data suggest that the enhanced release observed in *tom-1* mutants is due primarily to a prolongation of the evoked response.

Since evoked responses under our recording conditions largely reflect cholinergic transmission, we next asked whether the prolonged evoked response of *tom-1* mutants could be rescued by expressing TOM-1 protein specifically in cholinergic neurons. Recordings from *tom-1(nu468)* mutants possessing an integrated TOM-1A cDNA array ( *jaIs1052)* reduced the excitatory postsynaptic current amplitude when compared to both WT or *tom-1(nu468)* (1,094 ± 105 pA, *n* = 8, for *jaIs1052, p* ≤ 0.0006, relative to *tom-1(nu468))* and reversed the increased response duration to WT levels (4.7 ± 0.74 ms, *n* = 7, for *jaIs1052, p* = 0.0019, relative to *tom-1(nu468)),* resulting in a significant decrease in total charge integral (9.2 ± 1.3 pC, *n* = 7) relative to both WT ( *p* = 0.0001) and *tom-1(nu468)* ( *p* < 0.0001) ( Figure 2A and 2B). We attribute the decrease in release in *jaIs1052* relative to WT to overexpression of TOM-1A based on real-time PCR measurements of mRNA levels that indicate *tom-1A* mRNA levels are 11 ± 1.3 ( *n* = 3) times that of the WT. Together, these data suggest that TOM-1A is required presynaptically to regulate synaptic transmission. This observation is consistent with the neuron-specific expression pattern of TOM-1 based on a *tom-1* promoter::GFP fusion construct [ 16].

Although the TOM-1A expression experiments indicate that enhanced release in *tom-1* mutants has a presynaptic origin, the prolonged duration of the evoked response in *tom-1* mutants could be due to altered postsynaptic receptor kinetics. We therefore examined the electrophysiological properties of miniature postsynaptic events ( Figure 2C and 2D). Neither the decay rates (decay half-width: 2.05 ± 0.05 ms, *n* = 57, for WT vs. 2.03 ± 0.07 ms, *n* = 25, for *tom-1(ok285), p* > 0.5; and 2.12 ± 0.12 ms, *n* = 15, for *tom-1(nu468), p* > 0.8), the amplitude (24.7 ± 0.8 pA, *n* = 57, for WT vs. 22.4 ± 0.8 pA, *n* = 25, for *tom-1(ok285), p* = 0.1; and 26.8 ± 2.3 pA, *n* = 16, for *tom-1(nu468), p* = 0.28), nor the frequency (94.3 ± 6.6 Hz, *n* = 57, for WT vs. 88.4 ± 8.9 Hz, *n* = 26, for *tom-1(ok285), p* = 0.6; and 10.3 ± 13.5 Hz, *n* = 16, for *tom-1(nu468), p* = 0.6) ( Figure 2C and 2D) of miniature synaptic events in *tom-1* mutants were significantly different from the WT. These results suggest that the *tom-1* mutant synaptic phenotype is not due to changes in postsynaptic reception. Consistent with this conclusion, the miniature postsynaptic response kinetics of the neuronal TOM-1A–expressing integrants ( *jaIs1052)* (decay: 2.46 ± 0.2 ms, *n* = 7, *p* = 0.14; minifrequency: 84.8 ± 12.7 Hz, *n* = 8, *p* = 0.45) were not significantly different from *tom-1(nu468)* alone, despite the rescue of the evoked response duration and charge integral ( Figure 2C and 2D).

### Neuronal Architecture of *tom-1* Mutants

**Figure 3:**
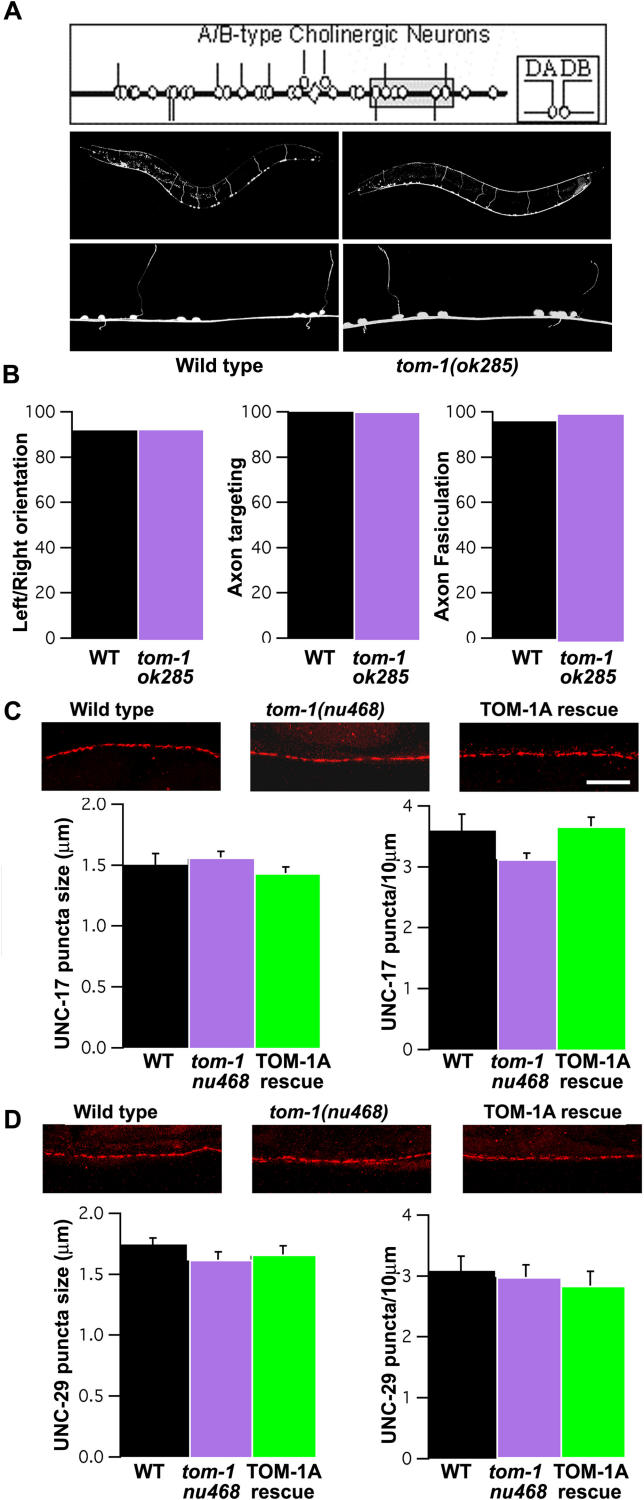
The Number and Distribution of NMJ Synapses in WT, *tom-1(nu468)–,* and TOM-1A–Expressing Animals (A) A schematic representation of the cholinergic motorneurons, and the outgrowth patterns of the dorsal neurons (inset). (B) The cholinergic motorneurons in *tom-1 (ok285)* animals (left) do not show any obvious changes from the wild type (right). (C) Representative images of the ventral nerve cord from WT, *tom-1(nu468),* and *tom-1(nu468*) mutants expressing TOM-1A ( *jaIs1052*) *,* immunolabeled with antibodies to the presynaptic marker, UNC-17 (vesicular ACh transporter). (D) UNC-17 punctum size (left) and the number of puncta per 10 μm (right) are normal in *tom-1* mutants and *jaIs1052*. (E) Representative images of the ventral nerve cord from WT, *tom-1(nu468),* and *jaIs1052* immunolabeled with antibodies to the postsynaptic marker, UNC-29 (ACh receptor). Scale bar = 10 micrometers (D) UNC-29 punctum size (μm) and the number of puncta per 10 μm are normal in *tom-1* mutants and *jaIs1052.* Data in (C) and (D) expressed as mean ± SEM.

Vertebrate tomosyn has been implicated in the regulation of neurite outgrowth in mammalian cultured neurons [ 19]. Therefore, we next addressed whether the enhanced evoked release in *C. elegans tom-1* mutants could be due to aberrant neuronal connectivity. To examine the neuronal cytoarchitecture of *tom-1* mutants, we crossed in an integrated array ( *juIs14*) expressing cytoplasmic green fluorescent protein (GFP) under the cholinergic neuron specific promoter *Pacr-2* [ 20]. Analysis of axonal left/right orientation, axon targeting, and axon fasciculation revealed no differences in the neuronal morphology of *tom-1(ok285);juIs14* relative to the WT ( Figure 3A and 3B), suggesting that the *tom-1* phenotype is not associated with any discernable innervation defects.

To test for possible changes in synaptogenesis, we examined the number and distribution of neuromuscular synapses in *tom-1* mutants. C. elegans neuromuscular synapses, which form en passant along the ventral nerve cord onto body wall muscles, can be visualized using pre- and postsynaptic markers. Specifically, synapses in WT and *tom-1* mutant animals were immunolabeled with antibodies to the presynaptic vesicular ACh transporter, UNC-17 [ 21] ( Figure 3C), and UNC-29, a postsynaptic muscle ACh receptor subunit ( Figure 3D). Analysis of the staining revealed that the number of synapses based on presynaptic staining (3.13 ± 0.1 UNC-17 puncta/10 μm, *n* = 5, for *tom-1(nu468)* vs. 3.6 ± 0.27 puncta/10 μm, *n* = 5, for WT, *p* = 0.14) and postsynaptic staining (2.89 ± 0.20 UNC-29 puncta/10 μm, *n* = 9, for *tom-1(nu468)* vs. 3.08 ± 0.24 puncta/10 μm, *n* = 10, for WT, *p* > 0.5) was not significantly altered. Similarly, the size of presynaptic puncta (1.55 ± 0.06 μm, *n* = 5, for *tom-1(nu468)* vs. 1.5 ± 0.1 μm, *n* = 5, for WT, *p* = 0.69) and postsynaptic puncta (1.61 ± 0.08 μm, *n* = 5, for *tom-1(nu468)* vs. 1.74 ± 0.07 μm, *n* = 5, for WT, *p* = 0.19) were not significantly affected in *tom-1(nu468*) *.* The reduction in evoked response in the TOM-1A–expressing strain ( *jaIs1052)* could also not be attributed to discernable changes in the number or size of cholinergic neuromuscular synapses ( Figure 3). These data suggest that the *tom-1* phenotype is not associated with any overt defects in morphogenesis of the nervous system and might therefore reflect a specific defect in synaptic function.

### Ultrastructural Analysis of *tom-1* Mutants

**Figure 4:**
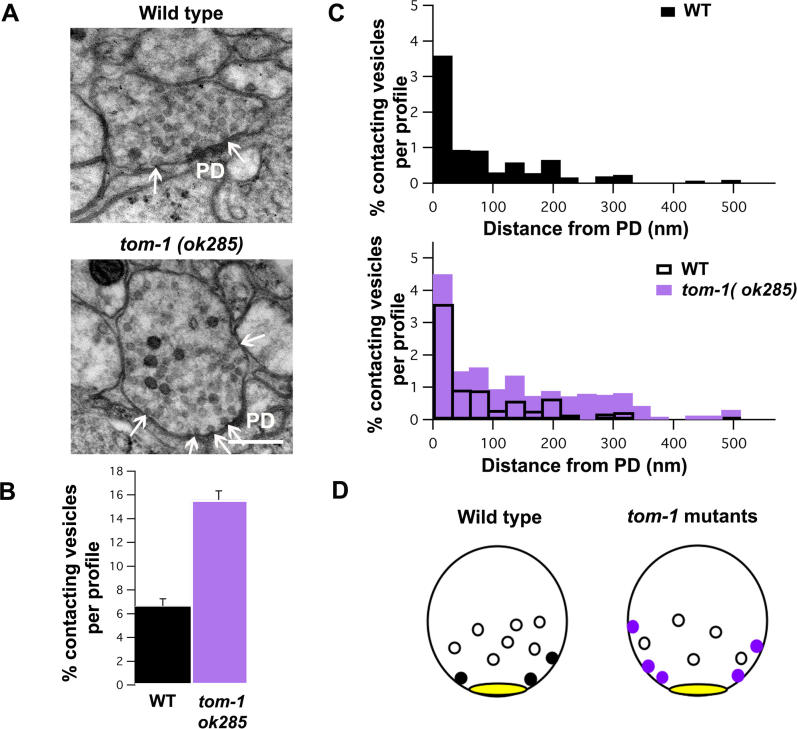
tom-1(ok285) Mutants Accumulate Vesicles That Are Contacting the Plasma Membrane (A) Examples of synaptic profiles from WT and *tom-1(ok285)* neuromuscular synapse prepared by high-pressure freeze and freeze substitution. The presynaptic density is labeled PD, and the plasma membrane-contacting vesicles are indicated with arrows. Scale bar, 100 nm. (B) Vesicles contacting the plasma membrane as a ratio of total vesicles per synaptic profile are increased in *tom-1(ok285)* mutant animals. (C) The distribution of plasma membrane-contacting vesicles relative to the presynaptic density, expressed in 30-nm bins as a ratio of total vesicles per profile for WT and *tom-1(ok285).* (D) Schematic representation of data in (C) depicting the distribution of vesicles contacting plasma membrane (black circles for WT, purple circles for *tom-1* mutants) relative to the presynaptic density. Data expressed as mean ± SEM.

To test whether the enhanced release in *tom-1* mutants resulted from increased synaptic vesicle biogenesis or altered vesicle distribution within synapses, we examined synaptic ultrastructure by electron microscopy (EM) ( Figure 4). Recent advances in high-pressure freeze and freeze substitution fixation techniques to prepare worms for EM have greatly improved the quality of ultrastructural data obtained in C. elegans [ 22]. The advantage of this technique over conventional fixation is that by virtue of the instantaneous freezing of the worms and gradual substitution with fixative, there is less fixation-induced osmotic shock that normally results in cell shrinkage and possible redistribution of synaptic vesicles to the plasma membrane. Using this technique, we found that while the average number of vesicles per synaptic profile did not differ from the WT in *tom-1(ok285)* mutants (17.45 ± 0.86 vesicles/profile, *n* = 86 profiles, for WT vs. 17 ± 0.81 vesicles/profile, *n* = 74 profiles, for *tom-1(ok285)),* vesicle localization was affected. Specifically, *tom-1(k285)* mutants exhibited a dramatic and significant ( *p* < 0.0001) increase in the number of vesicles contacting the plasma membrane (15.6% ± 0.75%, *n* = 74 synaptic profiles, for *tom-1(ok285)* vs. 8.5% ± 0.3%, *n* = 250 synaptic profiles, for WT) ( Figure 4A and 4B **).** In WT synapses, contacting vesicles were preferentially localized within ˜150 nm of the presynaptic density ( Figure 4C and 4D), whereas in *tom-1(ok285)* mutants the increase in membrane-contacting vesicles were distributed throughout the terminal ( Figure 4C and 4D).

**Figure 5:**
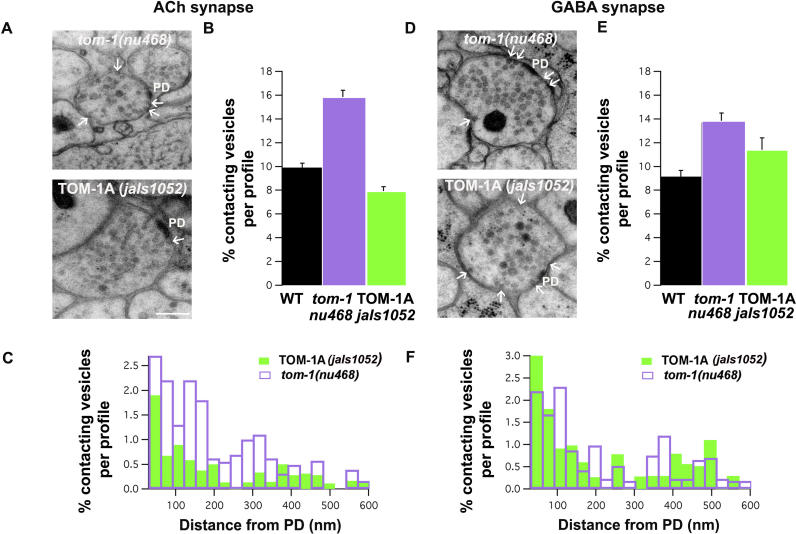
Comparison of the Plasma Membrane-Contacting Vesicles and Synaptic Vesicle Distribution in Cholinergic and GABAergic Synapses of WT, *tom-1(nu468),* and TOM-1A–Expressing Animals ( *jaIs1052*) (A) Representative images of cholinergic synapses in *tom-1(nu468)* (top) and *jaIs52* (bottom), Scale bar = 200 nm. (B) The ratio of the plasma membrane-contacting vesicles per profile is reduced in *tom-1(nu468*) mutants expressing TOM-1A in cholinergic neurons ( *jaIs1052)* compared to both *tom-1(nu468)* mutants and WT. (C) The distribution of plasma membrane-contacting vesicles relative to the presynaptic density in cholinergic synapses of *jaIs1052* and *tom-1(nu468)* animals, expressed in 30-nm bins as ratio of total vesicles per profile. (D) Representative images of a GABAergic synapse in *tom-1(nu468)* mutants (top) and *jaIs1052* (bottom). (E) The ratio of plasma membrane-contacting vesicles is significantly increased in *tom-1(nu468)* GABAergic synapses compared to WT, and is not rescued by expressing TOM-1A in cholinergic neurons. (F) The distribution of plasma membrane-contacting vesicles relative to the PD are similar in *tom-1(nu468)* and *jaIs1052* animals at GABAergic neuromuscular synapses. Data expressed as mean ± SEM.

We next examined the ultrastructure of *jaIs1052* ( Figure 5). Since TOM-1A expression was restricted to the cholinergic motor neurons of *tom-1(nu468)* mutants in *jaIs1052,* we compared the contacting vesicle pool of cholinergic synapses in *jaIs1052* versus *tom-1(nu468)* mutants ( Figure 5A and 5B). Consistent with the reduced evoked response, cholinergic synapses in *jaIs1052* had fewer contacting vesicles relative to *tom-1(nu468)* (7.9% ± 0.4% , *n* = 56 profiles, for *jaIs1052* vs. 15.8% ± 0.6 %, *n* = 49 profiles, for *tom-1(nu468), p* < 0.0001) ( Figure 5B). As an internal control for the specificity of the TOM-1A rescue, we also examined the number of contacting vesicles in the GABAergic synapses of *jaIs1052* worms ( Figure 5D and 5E). The number of contacting vesicles was not significantly different ( *p* > 0.5) between *jaIs1052* GABA synapses (11.4% ± 1%, *n* = 20) and *tom-1(nu468*) (13.7% ± 0.8 %, *n* = 19 profiles) ( Figure 5E). Similarly, the distribution of vesicles in cholinergic synapses was reduced throughout the terminal in *jaIs1052* cholinergic synapses ( Figure 5C), but not in the GABAergic synapses of the same worms ( Figure 5F). These data establish that vesicle contact is regulated by TOM-1 in both cholinergic and GABAergic synapses in *C. elegans.*

### Increased Vesicle Priming in *tom-1* Mutants

**Figure 6:**
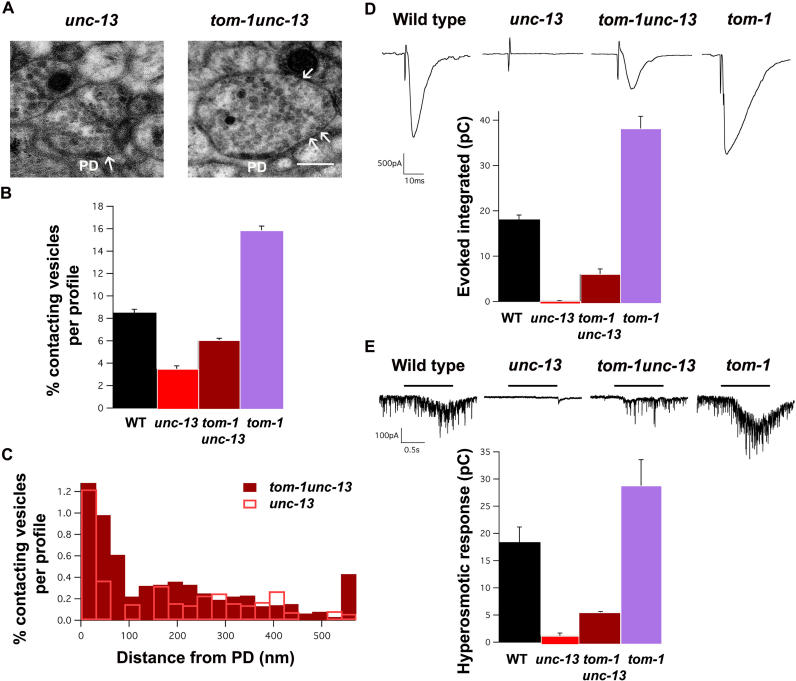
tom-1(ok285) Suppresses the Synaptic Defects of *unc-13(s69)* Mutants (A) Representative images of *unc-13(s69)* and *tom-1(ok285)unc13(s69).* (B) The ratio of plasma membrane-contacting vesicles per profile for WT (black), *unc-13(s69)* (red), *tom-1(ok285)unc13(s69)* (maroon), and *tom-1(ok285)* (purple) scale bar = 200 nm *.* (C) Comparison of *unc-13(s69)* and *tom-1(ok285)unc-13(s69)* plasma membrane-contacting vesicle distribution relative to the PD in 30-nm bins. (D) Representative NMJ recordings demonstrate that the evoked response absent in *unc-13(s69)* mutants is partially restored in *tom-1(ok285)unc13(s69)* double mutants. The average evoked charge integral of the *tom1 unc-13* double mutants is graphed relative to WT and *tom-1(ok285)* mutant responses **.** (E) Representative recordings of hyperosmotic responses demonstrate that the readily releasable pool of vesicles is increased in *tom-1(ok285)* relative to WT. The hyperosmotic response absent in *unc-13(s69)* mutants is also partially restored in *tom-1(ok285)unc13(s69)* double mutants. The mean total charge integral for synaptic events in the first second of the hyperosmotic response is graphed. Data expressed as mean ± SEM.

Since the formation of tomosyn SNARE complexes competes with the assembly of synaptobrevin-containing SNARE complexes, it has been proposed that tomosyn may inhibit synaptic vesicle priming. Therefore, the enhanced release and increased contacting vesicle pool in *tom-1* mutants could reflect an increase in the size of the readily releasable pool. To directly measure the size of the readily releasable pool in *tom-1* mutants, we recorded hyperosmotic responses at the neuromuscular junction. The hyperosmotic responses of both *tom-1* alleles were significantly increased relative to the WT (18.4 ± 2.8 pC, *n* = 13, for WT vs. 34.6 ± 5.1 pC, *n* = 6, for *tom-1(n468), p* = 0.0125; and 28.8 ± 4.8 pC, *n* = 10 for *tom-1(ok285), p* = 0.049) ( Figure 6), indicating that loss of TOM-1 results in an increased primed vesicle pool.

If the increased plasma membrane-contacting vesicle pool in *tom-1* mutants were a reflection of enhanced priming, we would predict that the priming-defective mutant *unc-13(s69)* would have fewer contacting vesicles. Morphometric analysis of *unc-13* mutants revealed a profound reduction in membrane-contacting vesicles relative to the WT (3.5% ± 0.3%, *n* = 101 synaptic profiles, for *unc-13(s69)* vs. 8.5% ± 0.3%, *n* = 250 synaptic profiles, for WT, *p* < 0.0001) ( Figure 6A and 6B). This reduction is consistent with the proposed role of UNC-13 in promoting SNARE complex formation and thus, vesicle apposition with the plasma membrane.

Our analysis of *tom-1* mutants suggests that priming is upregulated in the absence of TOM-1. Therefore, we next asked whether *tom-1* mutants could suppress the synaptic defects associated with *unc-13* mutants. In *tom-1(ok285) unc-13(s69)* double mutants the number of contacting vesicles relative to *unc-13(s69)* increased to 5.9% ± 0.2% per profile ( *n* = 124 synaptic profiles, *p* < 0.0001) ( Figure 6A and 6B). The distribution of contacting vesicles relative to the presynaptic density increased throughout the terminal in the *tom-1(ok285) unc-13(s69)* double mutants ( Figure 6C). Coincident with this morphological rescue, *tom-1(ok285) unc-13(s69)* double mutants exhibited increased aldicarb sensitivity relative to *unc-13(s69)* mutants, indicating that ACh release was partially restored (unpublished data). Recordings from the NMJ confirmed that both the evoked amplitude (3.3 ± 3.3 pA, *n* = 6, for *unc-13(s69)* vs. 752 ± 121 pA, *n* = 12, for *tom-1(ok285) unc-13(s69), p* < 0.0006), and total charge integral (0.11 ± 0.05 pC, *n* = 6, for *unc-13(s69)* vs. 6 ± 1.2 pC, *n* = 11, for *tom-1(ok285) unc-13(s69), p* < 0.0001) were increased in the double mutants with decay time constants similar to those of the WT (4.1 ± 0.8 ms, *n* = 11, for *tom-1(ok285) unc-13(s69)* vs. 4.6 ± 0.2 ms, *n* = 47, for WT, *p* > 0.5) ( Figure 6D). The recovery of synaptic function in *tom-1(ok285) unc-13(s69)* mutants was accompanied by a corresponding increase in the size of the hyperosmotic response (5.3 ± 0.34 pC, *n* = 3, for *tom-1(ok285) unc-13(s69),* vs. 1.17 ± 0.51 pC, *n* = 6, for *unc-13(s69), p* = 0.001) to 30% of the WT (17.4 ± 3.3 pC, *n* = 11, for WT) ( Figure 6E). Together, these data suggest that the priming defect of *unc-13* mutants can be partially ameliorated by removing TOM-1, further supporting the conclusion that TOM-1 functions to negatively regulate priming.

## Discussion

### Summary

We have examined the role of tomosyn in regulated synaptic transmission by analyzing two hypomorphic mutants of the C. elegans tomosyn homolog, *tom-1* [ 16]. Our results demonstrate that loss of TOM-1A and C isoforms enhances evoked release at the neuromuscular junction. In the absence of a complete *tom-1* null, the role of the remaining isoform, TOM-1B, remains to be elucidated. These results are consistent with a previously reported increase in sensitivity of *tom-1* mutants to the toxic effects of the acetylcholine esterase inhibitor, aldicarb, which is indicative of increased cholinergic transmission [ 16]. The enhanced neurotransmission observed electrophysiologically correlates with a redistribution of synaptic vesicles to the plasma membrane in *tom-1* mutant synapses. We further demonstrate that the ability of synaptic vesicles to contact the plasma membrane is regulated by the priming factor, UNC-13. When we generated *tom-1 unc-13* double mutants we observed partial suppression of the *unc-13* priming defect and concomitant restoration of the plasma membrane-contacting vesicle pool. Consistent with previous studies of dense core vesicle fusion [ 6, 10, 12, 13] and insulin release [ 14], these data suggest that tomosyn acts as a negative regulator of synaptic vesicle priming.

### What Is the Molecular Mechanism by which Tomosyn Negatively Regulates Vesicle Priming?

Priming is thought to involve formation of SNARE complexes between synaptobrevin, syntaxin, and SNAP-25, which bring the vesicle membrane into close apposition with the plasma membrane [ 5, 23]. Like vertebrate tomosyn [ 10], we demonstrate that the C-terminal of C. elegans TOM-1 also has the ability to form a complex with the SNARE domains of syntaxin and SNAP-25, suggesting this is a conserved tomosyn interaction. Vertebrate tomosyn competes with synaptobrevin for binding to syntaxin and SNAP-25 on the cytosolic surface of PC12 cell membrane sheets [ 10] as well as in in vitro biochemical studies [ 6]. By precluding vesicle-associated synaptobrevin from assembly into SNARE complexes, tomosyn is thus proposed to limit vesicle priming. This model is supported by several studies in which tomosyn overexpression has been shown to inhibit dense-core granule fusion [ 6, 10, 12] and neuronal exocytosis [ 15]. Furthermore, in chromaffin cells, tomosyn overexpression specifically inhibits the fast exocytotic burst corresponding to the fusion-competent primed granule pool [ 12]. In contrast to overexpression data, RNAi–mediated knockdown of tomosyn has produced mixed results. In cultured neurons tomosyn RNAi causes a reduction in synaptic vesicle release [ 15], where as RNAi in beta cells results in enhanced exocytosis [ 14]. This former study follows a previous report indicating that tomosyn RNAi profoundly inhibits directed neurite outgrowth, which could affect synaptogenesis under these culture conditions, resulting in fewer functional synapses [ 19]. In the context of an intact animal examined here, loss of tomosyn function does not appear to be deleterious for neurite outgrowth and synaptogenesis. Therefore, the enhanced neurotransmission we observe in *C. elegans tom-1* mutants is more consistent with RNAi in beta cells and appears to be attributable to functional changes in release properties rather than aberrant neuronal cytoarchitecture. Specifically, phenotypic analysis of *C. elegans tom-1* mutants supports the hypothesis that tomosyn acts as a negative regulator of synaptic vesicle priming in vivo.

In contrast to the *C. elegans tom-1* mutant ultrastructural phenotype, tomosyn overexpression in chromaffin cells was not associated with any changes in granule distribution based on conventional EM [ 12]. In TOM-1A–overexpressing synapses, we also observe near-normal numbers of contacting vesicles. Therefore, there is no conflict between the ultrastructural data reported for tomosyn overexpression and that of the present study. However, it remains to be seen whether loss of tomosyn in chromaffin cells is associated with increased granule association with the plasma membrane.

### Why Does *tom-1(ok285)* Suppress the Synaptic Defect of *unc-13(s69)?*

UNC-13 is a member of a conserved family of synaptic proteins implicated in vesicle priming [ 24– 26]. The mammalian homolog Munc13–1 is thought to interact with the N-terminus of syntaxin [ 27]. Syntaxin can adopt a closed state in solution that occludes the syntaxin SNARE domain (the H3 domain) required for SNARE complex formation [ 28]. UNC-13 binding to the N-terminus of syntaxin has been proposed to stabilize the syntaxin open configuration, increasing accessibility of the H3 domain [ 27]. Partial suppression of the *C. elegans unc-13* priming defect by overexpression of a constitutively open form of syntaxin supports this model [ 29]. Furthermore, mutations that disrupt UNC-13/syntaxin interactions in vitro have been shown to reduce release in both C. elegans and chromaffin cells [ 30, 31]. However, a Munc13 domain capable of partially rescuing priming in Munc13 KO mice, fails to bind syntaxin [ 32], suggesting alternative molecular mechanisms could also account for the priming function of Munc13. Here we show that *tom-1(ok285)* can also partially suppress the *unc-13(s69)* mutant phenotype. We postulate that in *unc-13* mutants, any transitions of syntaxin to the open state are prevented from forming functional SNARE complexes through the binding of tomosyn. It is also possible that the presence of UNC-13 normally excludes tomosyn from interacting with syntaxin and SNAP-25. In either case, we propose that in *tom-1 unc-13* double mutants, syntaxin has an increased probability of assembling into SNARE complexes because tomosyn no longer precludes synaptobrevin binding to the plasma membrane SNAREs.

### Why Is the Postsynaptic Response Prolonged in *C. elegans tom-1* Mutants?

Many of the contacting synaptic vesicles in *tom-1(ok285)* mutants are found distal to the presynaptic specialization. If, as at vertebrate NMJs [ 33], calcium channels are localized to the presynaptic specialization in *C. elegans,* calcium-triggered fusion of distal vesicles may be delayed relative to the proximal vesicles, resulting in a prolonged muscle response. It is also possible that the increased distance between distal neurotransmitter release sites and postsynaptic receptors prolongs the postsynaptic response in *tom-1* mutant muscles.

### How Might Tomosyn Restrict the Membrane Localization of Synaptic Vesicles?

One mechanism by which tomosyn may restrict synaptic vesicle fusion has recently emerged from the study of neurite outgrowth following tomosyn RNAi [ 19]. In cultured hippocampal neurons, knockdown of tomosyn increases neurite sprouting and branching while reducing neurite extension. This regulation is proposed to involve a high-affinity interaction between tomosyn and phosphorylated syntaxin (Ser14), which colocalize to the palms of growth cones [ 19]. The phosphorylation of syntaxin (Ser14p) by serine/threonine kinase (ROCK) leads to a five-fold increase in binding of tomosyn relative to synaptobrevin. Synaptobrevin-associated vesicles are thus predicted to be excluded from priming in growth cone palms, fusing instead at the leading edge of the growth cone, promoting neurite extension. Whether a similar mechanism functions to spatially restrict synaptic vesicle fusion to the active zone region in synaptic terminals remains to be investigated. However, syntaxin Ser14p is found throughout the rat cortex and appears to be excluded from regions rich in synaptic vesicles [ 34]. Therefore, it seems entirely possible that tomosyn may act to restrict exocytosis at synaptic terminals. Since syntaxin can be phosphorylated at Ser14 by both ROCK [ 19] and casein kinase II [ 34], this could also present a possible mechanism to regulate synaptic efficacy by affecting the level of tomosyn-mediated inhibition of vesicle priming.

The interaction between tomosyn and syntaxin is also regulated by protein kinase A–dependent phosphorylation of tomosyn in the variable linker between the WD40 domain and the C-terminal SNARE homology domain. Specifically, phosphorylation of tomosyn reduces the binding affinity of the tomosyn–syntaxin interaction [ 15]. Furthermore, protein kinase A–dependent synaptic facilitation appears to act in part through the phosphorylation of tomosyn, suggesting again that the interaction between tomosyn and syntaxin negatively regulates neurotransmitter release.

In summary, tomosyn interacts with syntaxin and SNAP-25 to form tomosyn SNARE complexes predicted to be nonfusogenic, which compete with SNARE complex formation and thus inhibit vesicle priming. Tomosyn perturbation analysis in C. elegans provides the first in vivo evidence in support of this model. Tomosyn mutants not only exhibit enhanced release but also suppress the priming defects of *unc-13* mutants. Future studies can now address whether the regulation of tomosyn function by kinases provide mechanisms for synaptic plasticity.

## Materials and Methods

### Genetics.

Nematodes were maintained on agar plates seeded with OP50 bacteria. Strains used were N2 Bristol, VC223 *tom-1(ok285),* BC168 *unc-13(s69),* SY1020 *tom-1(ok285)unc-13(s69),* SY1016 *tom-1(ok285)*; *oxIs12,* KP 3293 *tom-1(nu468),* and SY1181 *tom-1(nu468*); *jaIs1052* (integrated P *unc17*:: *tom1A*).

### In vitro biochemistry.

Purification of recombinant proteins SNAREs lacking TM domains. Vectors for C. elegans SNAP-25 and syntaxin were constructed as follows: the entire SNAP-25 coding region was inserted into pHO4d [ 35], the syntaxin cytoplasmic domain (aa 1–266) was cloned into pGEX-2T, and the SNB-1 cytoplasmic domain (aa 1–88) was cloned into pRSETC. The TOM-1 SNARE motif–coding region (TOM-1Ct; aa 1,137–1,211) was subcloned into pRSETC and purified as an N-terminally his6-tagged protein. Rat SNAP-25-His6 purified as described in [ 5] was provided by Phyllis Hanson (Washington University School of Medicine, Saint Louis, Missouri, United States). Proteins were purified using Ni-NTA resin or glutathione agarose, followed in some cases by further purification on a Mono S column using fast protein liquid chromatography.

Methodology. Fusion proteins were batch-purified on Ni-NTA–agarose according to the manufacturer's protocols (Qiagen, Valencia, California, United States) under native conditions with modified buffers. The lysis buffer consisted of 20 mM HEPES (pH 7.4), 500 mM KCl, 0.1 mM PMSF, 0.1% ßME, 5% glycerol; and 0–5 mM imidazole. The wash and elution buffer consisted of 20 mM HEPES (pH 7.9), 200 mM KCl, 0.1 mM PMSF, 0.1% ßME, 5% glycerol, 0.5% Triton X-100, and 1–300 mM imidazole. The dialysis buffer consisted of 10 mM HEPES (pH 7.9), 140 mM KCl, and 0.5% Triton X-100. The syntaxin-GST fusion protein and GST alone were batch-purified with glutathione agarose under native conditions [ 36]. The buffers used differed from those used in the Ni-NTA–agarose purifications only as follows: the lysis buffer contained no imidazole and 100 mM EDTA; the wash and elution buffer contained no imidazole, 2 mM EDTA, and 15 mM reduced glutathione. The purity of the fractions was verified by SDS-PAGE, and the concentration was determined by the Bradford assay.

TOM-1 complex formation assays. For in vitro complex formation, ˜1.6 μM of each of C. elegans syntaxin–GST (or GST), *rat* SNAP-25-His _6_, and C. elegans His _6_-TOM-1Ct (or -His _6_-SNB-1) were incubated from 2 h to overnight at 4 °C with rocking in D-buffer. The proteins were added to an aliquot of pre-equilibrated glutathione-agarose slurry and incubated at 4 °C for 1 h with rocking. The matrix was washed four times with 400 μl D-buffer. SDS–sample buffer was added, and half the reaction was heated (95 °C for 2 min) before analysis on SDS-PAGE. Proteins were detected by Coomassie Blue staining. D-buffer consisted of 10 mM HEPES (pH 7.9), 140 mM KCl, 0.5% Triton X-100, and 0.1 mM PMSF. Integrated densities were calculated using ImageJ (rsb.info.nih.gov/ij/). Complexes assembled using C. elegans SNAP-25-His _6_ were performed similarly, but after transfer to nitrocellulose, proteins were detected by immunoblotting using anti-GST, anti-His _6_ (SNAP-25, TOM-1Ct, and SNB-1), and anti-T7 (TOM-1Ct and SNB-1) antibodies.

### Behavioral assays.

Thrashing assays in M9 solution were performed by quantifying the number of body bends/min. For head tap responses, worms were allowed a 1-min settling period after placement in the assay chamber with food, before recording responses to a head tap for 20 s. Brood size was quantified by plating ten L4 hermaphrodites of each genotype onto individual plates and counting the number of progeny by removing hatched larvae and embryos for 3–4 d. Defecation rate was scored under a dissecting microscope using ten young adult worms for each genotype. For each worm, the duration of three consecutive defecations was measured and averaged. The cycle was defined as an interval between two expulsions of the gut content after visible contraction of the body wall muscles.

### Real-time PCR.

C. elegans total RNA was isolated using a Trizol reagent as described by the manufacturer (Invitrogen, Carlsbad, California, United States). Total RNA (0.5 μg) was used for reverse transcription using the SuperScript III First Strand Synthesis Kit (Invitrogen). Absence of genomic DNA was confirmed by PCR with primers designed to amplify the GFP coding region. GFP is a high copy coinjection marker, and we did not detect any genomic DNA contamination. For amplification of tomosyn isoform A, PCR primers were designed to amplify a unique exon. Dynamin was used as a reference for calibration. Real-time PCR was then performed by fluorescent detection and quantification of SYBR green-labeled PCR product using MJ Research Opticon2 real-time thermocycler (Bio-Rad, Hercules, California, United States). For quantification of relative initial transcript levels, the cycle threshold (C _t_) values for isoform A and a dynamin control were determined for each sample. The amount of *tom-1A* mRNA is reported as a ratio relative to the calibrator (dynamin). After RT-PCR, the products were subjected to agarose gel electrophoresis and sequencing to confirm the specificity of amplified products.

### Immunohistochemistry.

Immunohistochemistry was performed using a modified method of the whole worm fixation [ 37]. Briefly, the worms were frozen on dry ice between two slides, which were then split apart. Frozen animals were fixed in 4% slushy formaldehyde in PBS. Following fixation, animals were spun down at 1,600 rpm. Antibodies against UNC-29 (kindly provided by Dr. K. G. Miller) and UNC-17 (kindly provided by Dr. J. B. Rand) were used at a final dilution of 1:200 in PBS and 0.5% Triton X-100 with 3% BSA. Anti-rabbit and anti-mouse TRITC-conjugated secondary antibodies (Jackson ImmunoResearch Laboratories, West Grove, Pennsylvania, United States) were used at a 1:500 dilution for 4 h at 4 °C. Images were obtained with a 60× objective using an Olympus Optical (Tokyo, Japan) FV-500 laser-scanning confocal microscope. Puncta size and quantity were quantified from image projections of the ventral nerve cord region upstream of the vulva using ImageJ.

### Generation of TOM-1A rescuing lines.

*jaIs1052* strains expressing an integrated array of TOM-1A in *tom-1(nu468)* mutants were generated by irradiating animals expressing an extrachromasomal array of TOM-1A cDNA and a *Pmyo-2::gfp* coinjection marker under the cholinergic neuronal *unc-17* promoter (kindly provided by Dr. J. Kaplan) using a Cs source (total dose of 1,800 rad).

### Electrophysiology.

Electrophysiological methods were as previously described [ 25]. Briefly, animals were immobilized with cyanoacrylic glue, and a lateral cuticle incision was made exposing the ventral medial body wall muscles. Muscle recordings were made in the whole-cell voltage-clamp configuration (holding potential, −60 mV) using an EPC-10 patch-clamp amplifier and digitized at 2.9 kHz. The extracellular solution consisted of 150 mM NaCl, 5 mM KCl, 5 mM CaCl _2_, 4 mM MgCl _2_, 10 mM glucose, 5 mM sucrose, and 15 mM HEPES (pH 7.3, ˜340 mOsm). The patch pipette was filled with 120 mM KCl, 20 mM KOH, 4 mM MgCl _2_, 5 mM ( *N*-tris[Hydroxymethyl] methyl-2-aminoethane-sulfonic acid), 0.25 mM CaCl _2_, 4 mM Na ^2^ATP, 36 mM sucrose, and 5 mM EGTA (pH 7.2, ˜315 mOsm). Hyperosmotic data were recorded using an 800 mOsm extracellular solution achieved through addition of sucrose. Data were acquired using Pulse software (HEKA, Southboro, Massachusetts, United States) run on a Dell computer. Subsequent analysis and graphing was performed using Pulsefit (HEKA), Mini analysis (Synaptosoft Inc., Decatur, Georgia, United States) and Igor Pro (Wavemetrics, Lake Oswego, Oregon, United States).

### EM.

N2, *tom-1(ok285), tom-1(nu468), tom-1(ok285);unc-13(s69), unc-13(s69),* and TOM-1A rescue young-adult hermaphrodites were prepared for high-pressure freezing as previously described [ 22]. Briefly, ten to 15 animals were loaded in a specimen chamber filled with E. coli and immobilized by high-pressure freezing at ˜180 °C under high pressure in a Bal-Tec HPM010 and moved to liquid nitrogen.

Freeze substitution was performed in a Reichart AFS machine (Leica, Oberkochen, Germany) as previously described [ 38] using tannic acid (0.1%) and 0.5% gluteraldehyde fixative introduced over 4 d followed by 2% osmium. Fixed animals were then washed and embedded in Araldite 502 over a 48-h period at 60 °C.

Serial sections were cut at a thickness of 40–50 nm, collected on formvar-covered carbon coated copper grids (EMS, FCF2010-Cu), and counterstained in 2% or 2.5% aqueous uranyl acetate for 4 min, followed by Reynolds lead citrate for 2 min. Images were obtained on a JEOL JEM-1220 transmission electron microscope (JEOL, Tokyo, Japan) operating at 80 kV. Micrographs were collected using a Gatan digital camera (Pleasanton, California, United States).

Morphometric analysis of WT, *tom-1(ok285), tom-1(nu468), tom-1(ok285)unc-13(s69), unc-13(s69),* and ( *jaIs1052)* TOM-1A rescued animals were performed from ventral nerve cord serial sections. The analysis was performed blind. Images were quantified using NIH Image software. A synapse was defined as a set of serial sections containing a presynaptic specialization and two flanking sections from both sides without presynaptic specialization. Several morphometric measurements were obtained: the number of vesicles per profile, the distance from each vesicle membrane perpendicular to the plasma membrane, and the distance to the proximal edge of the presynaptic specialization.

## Supporting Information

Figure S1 Coomassie Blue–Stained PAGE Gel of Mixed-Species SNARE Complex Assembly Assays Complex assays were performed using C. elegans His _6_-tagged SNAREs and rat His _6_-tagged SNAREs. Purified syntaxin, SNAP-25, and synaptobrevin were mixed, incubated to allow complex formation, and separated on a PAGE gel to visualize complexes. One aliquot of each sample was loaded without heating (rt), and the other was heated to 95 °C for 5 min (b) prior to loading. The position of complexes, syntaxin, SNAP-25, and C. elegans synaptobrevin (which migrates just above the dye front) are labeled on the gel. Unfortunately, vertebrate (v) synaptobrevin migrates in the dye front and is not identifiable on the gel. Various combinations of C. elegans (c) and rat protein (v) mixes were tried. All reactions containing purified C. elegans SNAP-25 failed to form complexes efficiently, while all other combinations form complexes. The positions of molecular weight markers are on the left.
Methods: C. elegans His _6_-tagged SNAREs and rat SNAP-25 were purified as described in the main text. Rat His _6_-tagged synaptobrevin and syntaxin were a gift from Michael Crowder (Washington University School of Medicine) and purified as described in [ 39]. To form complexes, purified syntaxin, SNAP-25, and synaptobrevin were mixed, incubated overnight at 4° C, mixed with SDS-containing sample buffer, and run on a 4%–12% PAGE gel.
(292 KB JPG)

Figure S2 C. elegans TOM-1Ct Does Not Form a Stable Binary Complex with Syntaxin
A Coomassie-stained gel shows GST pull-down assays performed to eliminate the possibility that TOM-1Ct forms a complex with syntaxin in the absence of SNAP-25. C. elegans GST syntaxin or GST alone was incubated with either rat SNAP-25 (vSNAP-25) or C. elegans tomosyn C-terminal domain (TOM-1Ct). Glutathione agarose beads were added to collect complexes. The supernatant was removed, and the beads were washed extensively. Aliquots of the supernatant (sup) and the protein eluted from the glutathione agarose (beads) were separated on SDS-PAGE and stained for protein with Coomassie Blue. Although syntaxin bound to SNAP-25 in the absence of TOM-1Ct or synaptobrevin, TOM-1Ct did not interact with syntaxin in the absence of SNAP-25. Molecular weights of markers are labeled on the left.
Methods: proteins and GST pulled-down assays were performed as described in the main text.
(543 KB JPG)

Figure S3 Enlarged Micrographs of Representative NMJ Used in the Study (A) WT. (B) tom-1(ok285). (C) *tom-1(nu468)* cholinergic synapse. (D) Cholinergic synapse in *jaIs1052* (an integrated array of TOM-1A expressed in cholinergic neurons using the *acr-2* promoter, in a *tom-1(nu468)* mutant background). (E) *tom-1(nu468)* GABAergic synapse. (F) GABAergic synapse in *jaIs1052* (an integrated array of TOM-1A expressed in cholinergic neurons using the *acr-2* promoter, in a *tom-1(nu468)* mutant background). (G) *unc-13(s69).* (H) *tom-1(ok285) unc-13(s69)* double mutant. Arrows point to membrane-contacting synaptic vesicles.
(2.8 MB JPG)

### Accession Numbers

The Swiss-Prot/TrEMBL ( http://www.ebi.ac.uk/swissprot; http://www.ebi.ac.uk/embl) accession numbers for the following genes and gene products are syntaxin-1a (O35526), SNAP-25 (P60879), synaptobrevin-2 (a.k.a. VAMP-2) (P63044), tomosyn (Q8K400), synaptotagmin (P46096), Munc13 (Q9Z1N9), ROCK (P70335), Munc18 (Q548T0_MOUSE), *tom-1* (Q49HI2_CAEEL), TOM-1B (P91392_CAEEL), TOM-1A (P91389_CAEEL), TOM-1C (Q8T3B2_CAEEL), synaptobrevin (SNB-1) (O02495_CAEEL), syntaxin (UNC-64) (O16000_CAEEL), SNAP-25 (RIC-4) (O62414_CAEEL), and *unc-13* (P27715).

## Abbreviations

EM: electron microscopy
GFP: green fluorescent protein
NMJ: neuromuscular junction
RNAi: RNA interference
SNARE: soluble NSF attachment protein receptors
TOM-1Ct: C-terminal coiled-coil motif of TOM-1
WT: wild type
